# Physiological Characterisation of Human iPS-Derived Dopaminergic Neurons

**DOI:** 10.1371/journal.pone.0087388

**Published:** 2014-02-21

**Authors:** Elizabeth M. Hartfield, Michiko Yamasaki-Mann, Hugo J. Ribeiro Fernandes, Jane Vowles, William S. James, Sally A. Cowley, Richard Wade-Martins

**Affiliations:** 1 Oxford Parkinson’s Disease Centre, University of Oxford, Oxford, United Kingdom; 2 Department of Physiology, Anatomy and Genetics, University of Oxford, Oxford, United Kingdom; 3 The James Martin Stem Cell Facility, Sir William Dunn School of Pathology, University of Oxford, Oxford, United Kingdom; UCL Institute of Neurology, United Kingdom

## Abstract

Human induced pluripotent stem cells (hiPSCs) offer the potential to study otherwise inaccessible cell types. Critical to this is the directed differentiation of hiPSCs into functional cell lineages. This is of particular relevance to research into neurological disease, such as Parkinson’s disease (PD), in which midbrain dopaminergic neurons degenerate during disease progression but are unobtainable until post-mortem. Here we report a detailed study into the physiological maturation over time of human dopaminergic neurons *in vitro.* We first generated and differentiated hiPSC lines into midbrain dopaminergic neurons and performed a comprehensive characterisation to confirm dopaminergic functionality by demonstrating dopamine synthesis, release, and re-uptake. The neuronal cultures include cells positive for both tyrosine hydroxylase (TH) and G protein-activated inward rectifier potassium channel 2 (Kir3.2, henceforth referred to as GIRK2), representative of the A9 population of substantia nigra pars compacta (SNc) neurons vulnerable in PD. We observed for the first time the maturation of the slow autonomous pace-making (<10 Hz) and spontaneous synaptic activity typical of mature SNc dopaminergic neurons using a combination of calcium imaging and electrophysiology. hiPSC-derived neurons exhibited inositol tri-phosphate (IP3) receptor-dependent release of intracellular calcium from the endoplasmic reticulum in neuronal processes as calcium waves propagating from apical and distal dendrites, and in the soma. Finally, neurons were susceptible to the dopamine neuron-specific toxin 1-methyl-4-phenylpyridinium (MPP+) which reduced mitochondrial membrane potential and altered mitochondrial morphology. Mature hiPSC-derived dopaminergic neurons provide a neurophysiologically-defined model of previously inaccessible vulnerable SNc dopaminergic neurons to bridge the gap between clinical PD and animal models.

## Introduction

The midbrain dopaminergic (mDA) neurons can be divided into the substantia nigra pars compacta (SNc) and the ventral tegmental area (VTA). Neurons in these regions can be distinguished by pace-making activity that is driven by L-type calcium channels in the SNc and by sodium channels in the VTA [1], [2]. SNc neurons are typically studied in acute slice preparations of adult rodent brains and have a slow, spontaneous spike rate (2–5 Hz) which is accompanied by a sub-threshold oscillation [3], [4]. This characteristic firing pattern makes them easily identifiable within a mixed population of neurons. mDA neurons are large, have unmyelinated axons and each cell can give rise to thousands of synapses. Studies of rat nigrostriatal neurons have revealed cumulative axonal length that reaches up to 70 cm and that a single SNc neuron innervates up to 6% of the striatum [5], [6]. Such a cell is under extensive bioenergetic demand, including maintenance of membrane potential and propagation of action potentials, placing these cells under high levels of metabolic stress. However, it is not known to what extent these characteristic physiological properties contribute to the selective vulnerability of SNc dopaminergic neurons in the neurodegenerative disorder Parkinson’s disease (PD). Therefore, a representative neurophysiological model of human SNc dopaminergic neurons will be useful in studying the molecular processes underlying PD.

The generation of human induced pluripotent stem cells (hiPSCs) from somatic cells is enabled by the advent of a range of reprogramming technologies which express pluripotency factors (OCT4, SOX2, KLF4, c-Myc, NANOG and LIN28) [7], [8]. We have utilised this technology and reprogrammed iPSCs from normal human dermal fibroblasts (NHDFs). Characterisation of iPSC lines is technically challenging and time consuming. Here we have improved upon some of the original methods of iPSC characterisation and applied two high-throughput methods of characterisation of iPSC lines: single polymorphic nucleotide (SNP) profiling for karyotyping (Illumina CytoSNP-12-v2.0 array) and transcriptomic analysis for pluripotency (PluriTest). These are faster, more economical and more accessible techniques than the traditional methods for karyotyping (Fluorescent *In Situ* Hybridisation of metaphase spreads) and pluripotency (teratoma formation in immunosuppressed mice).

The advantage of hiPSCs is that they can be generated from patients carrying disease-associated mutations, therefore the differentiation of these cells into a specific cell type would allow the study of disease in its correct cellular context. Several studies have utilised Parkinson’s disease (PD) patient iPSCs and are beginning to show disease-specific phenotypes in differentiated dopaminergic (DA) neurons [9]–[13]. However, it is not clear that these differentiated DA neurons accurately model the neurophysiology of the SNc midbrain DA neuron vulnerable in PD.

Here we report a detailed study into the physiology of human dopaminergic neuronal cultures as they mature *in vitro.* We first performed a comprehensive characterisation of differentiated mDA neurons to demonstrate DA functionality by dopamine synthesis, release and re-uptake. We used a combination of electrophysiology and calcium imaging to monitor maturation of the spontaneous slow pace-making and synaptic activity at <10 Hz typical of mature midbrain dopaminergic neurons. Neuronal cultures include cells positive for both tyrosine hydroxylase (TH) and G protein-activated inward rectifier potassium channel 2 (Kir3.2, henceforth referred to as GIRK2), representative of the vulnerable A9 population of substantia nigra neurons in PD. These neurons provide a high-quality model of vulnerable SNc human dopaminergic neurons and bridge the gap between clinical PD and animal models of dopaminergic systems.

## Materials and Methods

### Ethics Statement

Normal human dermal fibroblasts used for reprogramming to pluripotency were purchased from Lonza, who provide the following ethics statement: ‘These cells were isolated from donated human tissue after obtaining permission for their use in research applications by informed consent or legal authorization.’ The human hiPS cell lines derived from these fibroblasts were generated as control lines for part of a larger-scale project (Ethics committee: National Health Service, Health Research Authority, NRES Committee South Central – Berkshire, UK, who specifically approved this part of the study - REC 10/H0505/71).

### Generation and Culture of hiPSC Lines

iPS-NHDF-1 and iPS-NHDF-2 were derived from NHDFs (Lonza; CC-2511). Reprogramming plasmids were obtained from Addgene (17220: pMXs-hc-MYC, 17219: pMXs-hKLF4, 17218: pMXs-hSOX2, 17217: pMXs-hOCT3/4, 13354: pMXs-Nanog) [14] and packaged using the Plat-GP retroviral packaging cell line. Fibroblasts were infected on days 0 and 1with 5 µg/ml polybrene and spinoculation (1200×g for 45 minutes at 16°C). They were transferred onto mitomycin C-inactivated mouse embryonic feeder cells (MEFs; outbred Swiss mice [15], [16] established and maintained at the Department of Pathology, Oxford) on 0.1% gelatin coated plates on day 4. From day 5 onwards, cells were cultured in KnockOut™ serum replacement medium (Life Technologies) supplemented with 50 µg/ml ascorbic acid and 0.5 µM valproic acid. Medium was replaced (50%) on alternate days, and substituted with MEF-conditioned medium from day 10 onwards.

Colonies displaying iPSC morphology were picked on day 28 and transferred onto MEFs by manual dissection every 5–7 days. Prior to differentiation, iPSC lines were adapted to feeder-free conditions onto Matrigel-coated plates (BD Matrigel hESC-qualified Matrix) in mTeSR™1 (StemCell Technologies) supplemented with Rock inhibitor Y27632 (10 µM; Calbiochem) on the day of passage.

### Assessment of Genome Integrity

Genome integrity was assessed by an Illumina Human CytoSNP-12v2.1 beadchip array (∼300,000 markers) and analysed using KaryoStudio software (Illumina). The iPSC lines were also subjected to M-FISH analysis of metaphase chromosomal spreads, as described previously [17]. Multiple metaphases (>30) per iPSC line were assessed.

### PluriTest

RNA was extracted from iPS-NHDF-, iPS-NHDF-2 and iPS-DF19.9.11TH using an RNeasy kit (Qiagen) for Illumina HT12v4 transcriptome array analysis. The data files were then uploaded to www.pluritest.org and scored for pluripotency, as previously described [18].

### Generation of Embryoid Bodies

Feeder-free iPSCs were dissociated with TryplE and seeded into Aggrewell plates (10,000 cells per EB; Stem Cell Technologies) in mTeSR™-1 medium supplemented with Y27632 (10 µM). EBs were harvested after 4 days (75% daily mTeSR-1 medium change) and differentiated as appropriate.

### Differentiation to 3 Germ Layers

Embryoid bodies were directed to neurectoderm by culture on gelatin-coated coverslips in dopaminergic differentiation medium (described below). Mesodermal cells were differentiated in EB media (KO-DMEM supplemented with 2 mM glutamax, 1% non-essential amino acids, 55 µM 2-mercaptoethanol, 0.5 mM ascorbic acid, 100 U/ml penicillin/100 µg/ml streptomycin, and foetal calf serum (10% [v/v]; HyClone). Endoderm was differentiated as for mesoderm but without ascorbic acid.

### Differentiation into Midbrain Dopaminergic Neurons

All materials obtained from Life Technologies unless otherwise stated. Briefly, EBs were plated onto Geltrex-coated plates in Neural Induction medium 1 (DMEM/F12 supplemented with L-glutamine [2 mM], N2 supplement, bovine serum albumin [1 mg/ml], Y27632 [10 µM; Tocris], SB431542 [10 µM, Tocris], noggin [200 ng/ml] and antibiotic/antimycotic [1% v/v]). After 4 days, medium was changed to Neural Induction medium 2 (as NI1, without SB431542 and noggin, with the addition of sonic hedgehog [SHH C24II], 200 ng/ml; R&D Systems) and incubated for 6 days. Medium was then additionally supplemented with fibroblast growth factor-8a (FGF8a, 100 ng/ml; R&D Systems), heparin (5 µg/ml; Sigma), ascorbic acid (200 µM; Sigma) and brain derived neurotrophic factor (BDNF, 20 ng/ml) and maintained for 7–14 days, depending upon appearance of neural rosette structures. Neural progenitor cells were manually passaged and replated onto poly-D-lysine/laminin-coated plates in final differentiation medium (DMEM/F12 supplemented with L-glutamine [2 mM], N2 supplement, BDNF [20 µg/ml], glial-derived neurotrophic factor [GDNF, 20 µg/ml], N^6^,2′-O-dibutyryladenosine 3′,5′-cyclic monophosphate sodium salt [dCAMP, 0.5 mM; Sigma], laminin [1 µg/ml] and antibiotic/antimycotic (1% [v/v]). Neurons were matured for a further 2–6 weeks in this medium (total time in culture 4–10 weeks) before experimental procedures were carried out. Total time in culture from the initiation of neuralisation (EB stage) is 4–10 weeks.

### RT-PCR Analysis

RNA was extracted from cells using Trizol (Life Technologies) and purified using the RNeasy kit (QIAGEN). Reverse transcription was performed using Superscript III (Life Technologies) according to manufacturer’s instructions. Polymerase chain reactions (PCR) were set up as follows: cDNA (25 ng), AmpliTaq Gold 10x buffer (Applied Biosystems), MgCl_2_ (1.5 mM), forward and reverse primers (1 µM each), dNTP mix (0.5 mM; Life Technologies) and 1 U Taq polymerase (AmpliTaq Gold, Applied Biosystems). Primer sequences are detailed in Table S1.

### Immunocytochemistry

Cells were fixed in 4% paraformaldehyde and permeabilised in 0.1% Triton-X100 prior to immunostaining, except in the case of dopamine staining where cells were fixed according to the following protocol: Cells were washed in ‘pre-fix solution’ (0.1 M cacodylate and 1% sodium metabisulphite [w/v], pH 6.2) then fixed in 3% [v/v] glutaraldehyde solution (pH 7.5) for 15 minutes. Coverslips were then washed 3 times in ‘post-fix solution’ (0.05 M tris and 0.85% [w/v] sodium metabisulfite, pH 7.5). A reduction step was carried out in 0.1 M sodium borohydride for 10 minutes prior to following the conventional immunostaining protocol. Briefly, coverslips were blocked in 10% goat or donkey serum for 1 hour before incubating with primary antibodies overnight at 4°C. Antibodies used as follows: α-sarcomeric actin (Sigma), FOXA2 (1∶250; R&D systems), Tuj1 (1∶500; Covance), TH (1∶500; Millipore), α-synuclein (1∶500; BD biosciences), LRRK2 (1∶1000; Epitomics), Tau (1∶700, a kind gift from Peter Davies), dopamine (1∶500, Abcam), SV2B (1∶200, Synaptic Systems), IP_3_/BM (a generous gift from Dr Stuart Conway, University of Oxford), DAT (1∶500; Alpha Diagnositcs), GIRK2 (1∶200; Abcam), VMAT2 (1∶500, Chemicon), Glucocerobrosidase (1∶1000, Abcam), PITX3 (1∶100, Life Technologies) and NURR1 (1∶400, Millipore). Secondary antibodies (Alexa fluor, Life Technologies) were incubated for 1 hour at room temperature before mounting and analysis. Images were captured on the Leica SP5 confocal microscope. For quantification, multiple random fields (5–10 per coverslip) were imaged from at least 3 independent differentiations.

### Western Blot Analysis

Western blotting was carried out on whole cell lysates that had been extracted using RIPA buffer (tris [50 mM, pH 8], sodium chloride [150 mM], sodium dodecyl sulphate [SDS; 0.1% w/v], sodium deoxycholate [0.5% w/v] and nonidet-P40 [1% w/v]). Before loading, samples were denatured. Protein separation was achieved using SDS polyacrylamide gel electrophoresis and transferred onto PVDF membrane. Antibodies used as follows: TH (1∶500; Millipore), Dopa decarboxylase (1∶1000; Millipore), DAT (1∶1000; Thermoscientific).

### High Performance Liquid Chromatography (HPLC)

Dopamine content was analysed using HPLC with electrochemical detection. Differentiated neurons were harvested in 0.1 M perchloric acid. Lysate was filtered and injected into the HPLC system via an autosampler (Jasco) and monoamines separated on a 250 mm Microsorb C18 reverse-phase column and detected with an LC-4B electrochemical detector (Decade SDC, Antec). The mobile phase consisted of methanol (13% v/v), NaH_2_PO_4_ (120 mM), EDTA (0.8 mM), and sodium octane sulfonate (3.2 mM) at pH 3.27. The flow rate was fixed at 1 ml/min and dopamine content determined by comparing samples to standard solutions. Corresponding dopamine content normalised to protein.

### DAT Function

Functional dopamine active transporter (DAT) activity was quantified by incubating differentiated cultures in the presence of ^3^H-DA (10 nM, GE Healthcare). Briefly, neurons were washed once with PBS prior to addition of ^3^H-DA either in the presence or absence of mazindol (10 µM, Sigma). For each experimental well, 1 ml of 3H-DA mixture was added for the required time before removal. Uptake was stopped by the addition of ice-cold PBS followed by lysis in sodium hydroxide (1 M). A small sample was retained for protein analysis whilst the remainder was diluted with 5 ml scintillation fluid (OptiPhase SuperMix, Perkin-Elmer) and quantified using a scintillation counter (Beckman Coulter).

### Whole Cell Patch Clamp Recordings

Electrophysiological experiments were performed on 6–10 week differentiated neurons at 32°C. Intracellular recordings were obtained using Axon Instruments Multiclamp 700B amplifier and digitized at 10–20 kHz (Digidata 1440A). Extracellular solution was as follows (mM): 129 NaCl, 5 KCl, 2 CaCl_2_, 1 MgCl_2_, 30 Glucose, 25 HEPES, pH 7.4. Whole-cell patch-clamp electrodes (4–7 MΩ) were filled with 120 mM K D-gluconate, 25 mM KCl, 4 mM MgATP, 2 mM NaGTP, 4 mM Na2-phospho-creatin, 10 mM EGTA, 1 mM CaCl2 and 10 mM HEPES (pH 7.4 with KCl at 25°C). Biocytin (0.1%) was added to the pipette solution to mark the recorded neuron for later cytochemical characterization. Spontaneous postsynaptic currents measured under this experimental condition at −70 mV are a superposition of mEPSCs. Further analysis and statistics were performed using IgorPRO (Wavemetrics) with custom-written scripts.

### Ca^2+^ Imaging

Neurons were loaded with Fluo 4-AM (5 µM, Life Technologies) for 60 min in HBSS supplemented with 10 mM HEPES. Fluorescence microscopy was performed using a Nikon TE 2000 inverted microscope and an EMCCD camera (Evolve 128, Photometrics) imaging fluorescence emission (510–600 nm) at a frame rate of 100 s^−1^ at 37°C. Fluorescence measurements are expressed as a ratio (ΔF/F_o_) of the mean change in fluorescence (ΔF) relative to the resting fluorescence before stimulation (F_o_). Mean values of F_o_ were obtained by averaging over several scans/frames before stimulation.

### Measurements of Mitochondrial Membrane Potentials (ΔΨ)

Mitochondrial ΔΨ was measured by loading cells with 20 nM tetramethyl rhodamine methyl ester (TMRM, Life Technologies) for 30 min at 37°C. Images were taken by the same inverted microscope described above equipped with objective lenses (20x, N.A. 0.75 and 40x, N.A. 1.30). TMRM excitation (560 nm) and emission (590–650 nm) was measured. Carbonyl cyanide p-trifluoromethoxyphenylhydrazone (CCCP (10 µM; Life Technologies) was added to collapse membrane potential.

## Results

### hiPSC Generation and Characterisation

**Figure 1:**
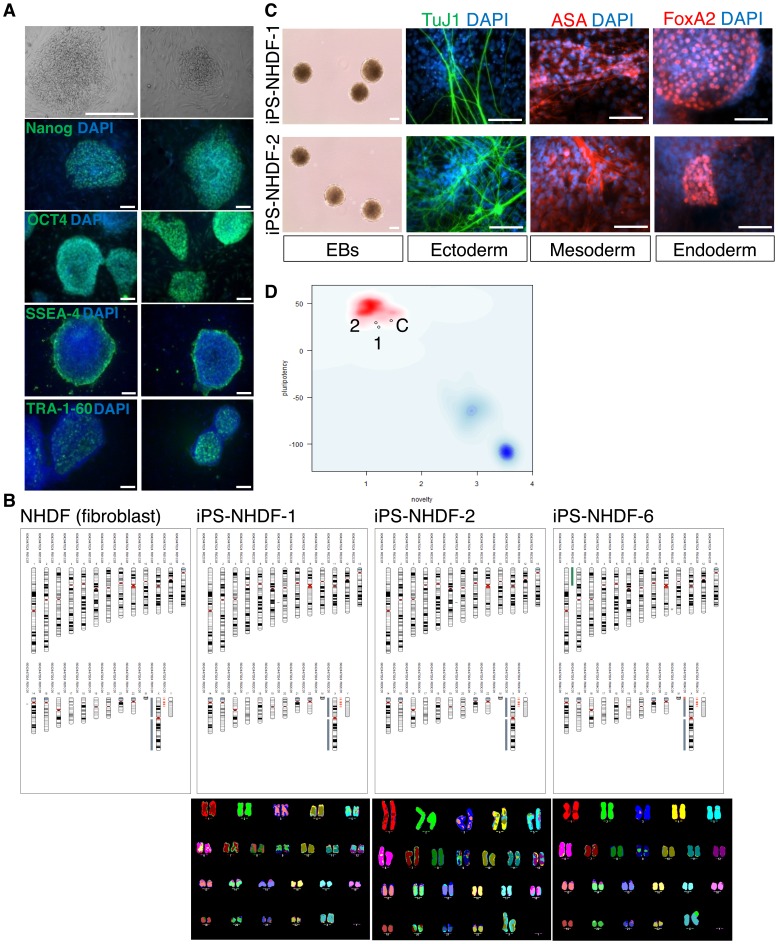
Characterisation of hiPSC lines derived from normal dermal human fibroblasts. A) Two hiPSC lines were selected for in-depth analyses, NHDF-1 and NHDF-2. These reprogrammed colonies showed hESC-like morphology, for example, tightly packed colonies with high nucleus to cytoplasm ratio (top row). These colonies expressed pluripotency markers hNanog, hOCT4, SSEA-4 and TRA-1-60. Scale bar: 100 µm. B) Genome integrity analysis was performed using both the Illumina Human CytoSNP-12 beadchip with analysis using Karyostudio and using M-FISH. The top panel shows the Illumina analysis of three derived hiPSC clones (iPS-NHDF-1, -2 and -6), using the parental fibroblasts as reference (NHDF). The two analyses indicated a normal chromosomal complement and no gross structural abnormalities for iPS-NHDF-1 and iPS-NHDF-2, but iPS-NHDF-6 showed a translocation and amplification of part of chromosome 2, t(X:2), and was not used in further experiments. GEO: GSE43904. C) iPS-NHDF-1 and iPS-NHDF-2 cell lines are competent at differentiation to three germ layers *in vitro*. Left column shows embryoid body (EB) formation 4 days after forced aggregation of hiPSCs using Aggrewells. Second column, directed differentiation to neuroectoderm, showing neurons stained for βIII tubulin, using a TujI antibody. Third column, directed differentiation to mesoderm, showing cells stained with antibody against α-sarcomeric actin (ASA). Fourth column, directed differentiation to endoderm, showing nuclei stained with an antibody against the transcription factor, FoxA2. Nuclei are counterstained with DAPI (blue). Scale bar:100 µm. D) PluriTest analysis of Illumina HT12v4 transcriptome array data shows iPS-NHDF-1 and iPS-NHDF-2 to cluster with pluripotent stem cells (red cloud) and not with partly- or differentiated cells (blue clouds). Each circle represents one iPSC line: 1 = iPS-NHDF-1; 2 = iPS-NHDF-2; C = control iPSC line iPS-DF19.9.11TH. GEO: GSE43904.

NHDFs were reprogrammed by retroviral delivery of *hOCT4*, *hSOX2*, *hKLF4*, *hc-MYC* and *mNanog*, in the presence of enhancer molecules. Reprogramming resulted in 6 morphologically acceptable clones, of which iPSC lines NHDF-1, -2 and -6 passaged well and were selected for assessment of genome integrity. These colonies were positive for the pluripotency marker TRA antigen, human Nanog, Oct4, SSEA-4 and TRA-1-60 by immunocytochemistry (Figure 1A). They also expressed transcripts for endogenous pluripotency genes, and exogenous reprogramming genes were silenced as detected by semi-quantitative RT-PCR ().

Genome integrity was assessed using two different methods: M-FISH and Illumina CytoSNP-12-v2.0 array (Figure 1B). Analysis of >30 metaphases by M-FISH indicated that iPS-NHDF-1 and-2 were of normal karyotype. However, iPS-NHDF-6 appeared to have a clonal translocation (t[X:2]). The HumanCytoSNP-12 BeadChip contains nearly 300,000 genetic markers and as such provides very detailed resolution of the integrity of the genome compared with M-FISH. Analysis by CytoSNP array revealed the apparent translocation in iPS-NHDF-6 to also be an amplification of part of the short arm of chromosome 2 (indicated by green band in Figure 1B), together with a deletion (in red) of the end of the short arm of the X chromosome (see also Table S2). In short, the genetic lesion was detectable by both methods, but neither gave complete information. iPS-NHDF-1 and -2 were classified as karyotypically normal by both methods, so were used for further analyses and downstream experiments.

We next sought to assess pluripotency in these cell lines and began with *in vitro* differentiation into cell types representative of the 3 germ layers. Cells differentiated into embryoid bodies (EBs) in Aggrewells (Figure 1C, left panel) were competent at directed differentiation to all three germline lineages, as assessed by expression of lineage-specific markers: Tuj1 (neurectoderm), α-sarcomeric actin (mesoderm), and FoxA2 (endoderm) (Figure 1C).

A more quantitative assessment of pluripotency has recently been developed based upon analysis of transcriptome data from iPSCs and comparing this to a large reference set of genome-wide profiles from multiple cell and tissue types [18]. We therefore subjected iPS-NHDF-1 and-2 (referred to as ‘1’ and ‘2’, respectively, in Figure 1D) to this analysis, alongside a previously characterised iPSC line, iPS-DF19.9.11TH (referred to as ‘C’ [Control] in Figure 1D) [19]. Both iPS-NHDF-1 and -2, and iPS-DF19.9.11TH, were deemed to be fully reprogrammed and pluripotent (Figure 1D). The PluriTest measures a ‘pluripotency score’ (expression of pluripotency genes, with high scores correlating with high pluripotency; iPS-NHDF-1: 25.27, iPS-NHDF-2: 29.51 and iPS-DH19.9.11TH: 31.73) and a ‘novelty’ score (expression of non-pluripotent stem cell genes, therefore this score inversely correlates with pluripotency: iPS-NHDF-1: 1.23, iPS-NHDF-2: 1.18 and iPS-DH19.9.11TH: 1.46).

The Illumina HT12v4 transcriptome array results have been deposited in the Gene Expression Omnibus (GEO) under accession number GSE43903. SNP datasets have been deposited in GEO under the accession number GSE43902. These are both contained in the Superseries GSE43904.

### Differentiation of Midbrain Dopaminergic Neurons

**Figure 2:**
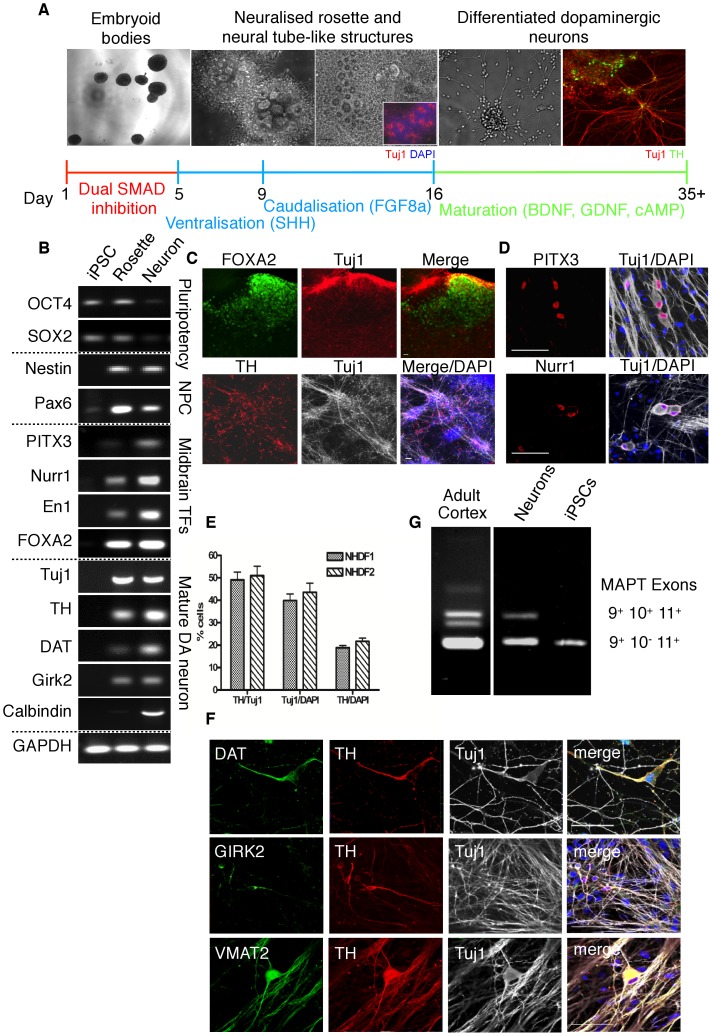
Differentiation of human dopaminergic neurons. A) hiPS cells were directed to differentiate into dopaminergic neurons. Following midbrain patterning, neural progenitor cells were manually passaged (day 16+). Dopaminergic neurons were matured for a total of 6–10 weeks before experimentation. B) RT-PCR analysis of gene expression in cells at different stages of differentiation (EB: day 0, Rosette: day 16, Neuron: day 35). Pluripotency genes (OCT4 and SOX2) were down-regulated as cells differentiate and concurrently midbrain dopaminergic gene expression was upregulated. Midbrain dopaminergic neuronal markers (PITX3, NURR1, EN1, TH and GIRK2) were strongly expressed in differentiated neuronal populations. C) Immunocytochemical staining for TH (bottom panels) revealed an abundant population of neurons. Large clusters of neurons expressed the floorplate marker FOXA2 (top panels). Scale bar: 70 µm. D) The midbrain transcription factors, PITX3 and NURR1 were expressed in differentiated neurons. Scale bar: 70 µm. E) Efficiency of dopaminergic differentiation was quantified from at least 3 independent differentiations. iPS- NHDF1 and 2 lines differentiated with similar efficiencies into neurons (39.59% ±3.56 and 41.73% ±2.02, expressed as Tuj1/DAPI). The proportion of neurons that were dopaminergic was also similar (NHDF1: 50.35% ±3.4 and NHDF2: 52.54% ±2.76). The total number of cells that were TH^+^ were 19.21% ±2.05 and 21.5% ±1.53 for NHDF1 and NHDF2, respectively. Data are expressed as the mean ± SEM. F) Co-labelling of differentiated neurons revealed expression of dopaminergic neuronal proteins such as DAT and VMAT2 together with TH. The A9 dopaminergic neuron marker, GIRK2 was also expressed in some TH-positive cells. Scale bar: 70 µm. G) Analysis of MAPT exon splicing indicated that differentiated neurons have a splicing pattern similar to that of the human adult cortex. Exon skipping of exons 2, 3 and 10 was observed in undifferentiated hiPSCs, which is typical of embryonic neural tissues.

hiPSCs were directed to differentiate into mDA neurons by first plating EBs onto tissue culture plates (Day 0) in the presence of Rock inhibitor (Y27632) (Figure 2A). We had previously observed that Y27632 substantially increased the size of neural progenitor cell colonies derived from human embryonic stem cells (hESCs; Figure S2), therefore Y27632 was used to supplement the culture medium throughout the differentiation process. Midbrain DA neuron differentiation was developed through a combination of previously published protocols. Neural patterning was initiated by a dual SMAD inhibition strategy [20] and specification towards a ventral mDA fate was performed using the morphogens sonic hedgehog (SHH) and fibroblast growth factor-8a [21] (FGF8a) (Figure 2A). Neuronal maturation was achieved through ascorbic acid, BDNF, GDNF and cAMP [20]–[22]. RT-PCR was performed at different stages of differentiation (Figure 2B) and showed that the endogenous pluripotency genes *OCT4* and *SOX2* were down-regulated in differentiated neurons. Concurrently, neuronal progenitor genes (*NESTIN* and *PAX6*) were upregulated in progenitor cells at the rosette stage, therefore neural patterning had successfully been initiated. Using a panel of genes we observed gene expression in differentiated neuronal cultures typical of midbrain dopaminergic neurons. The midbrain transcription factors *ENGRAILED* (*EN1*), *FOXA2*, calbindin, *PITX3* and *NURR1* were expressed in differentiated neurons. Extensive immunocytochemical analysis was performed on differentiated neurons and large populations of tyrosine hydroxylase- (TH) expressing cells were found (Figure 2C). In addition, clusters of differentiated neurons expressed FOXA2, suggesting that these were ventral midbrain-derived neurons. FOXA2+ cells were quantified in our differentiated cultures and we found ∼30% of TH+ cells total expressed this protein. (Figure S3). Furthermore, PITX3 and NURR1 proteins were also detected, with expected nuclear localisation (Figure 2D).

Differentiation efficiency was quantified by cell counting (Figure 2E). Both cell lines differentiated with similar efficiency into neurons (NHDF1: 39.59% ±3.56; NHDF2: 41.73% ±2.023, expressed as a percentage of total cells). Similarly, both cell lines produced equivalent levels of TH^+^ cells (NHDF1: 19.12% ±2.05; NHDF2: 21.5% ±1.53, as a percentage of total cells). Approximately half of the differentiated neurons were TH^+^ (NHDF1: 50.35% ±3.4; NHDF2: 52.54% ±1.53). Therefore, a high proportion of dopaminergic neurons were obtained.

In addition, the dopaminergic genes *TH* and *DAT* were expressed in increasing amounts as differentiation proceeded. Of particular note is the expression of the GIRK2 gene and protein, which co-localised with TH-expressing cells (Figure 2F). This suggests that the differentiated population contained A9 dopaminergic neurons typical of the substantia nigra pars compacta (SNc), which are vulnerable in PD [23]. Quantification of neuronal cultures revealed that 32.5% of cells expressed GIRK2, and that 53.5% of TH+ neurons expressed GIRK2, therefore a substantial population were of the A9 phenotype (Figure S3). Further analyses showed that mature TH^+^ neurons also expressed vesicular monoamine transporter 2 (VMAT2), involved in sequestration of monoamines into synaptic vesicles (Figure 2F).

Alternative splicing of *MAPT* exon 10 is under exquisite developmental control and can be used as an indicator of maturity of human neurons as development proceeds [24]. Undifferentiated hiPSCs express exclusively the shorter exon 10^−^ isoform, whereas differentiated neurons express the exon 10^+^ isoform similar to that of adult human neurons from post-mortem brain (Figure 2G).

We examined the expression of several proteins implicated in the pathology of PD, including leucine rich repeat kinase 2 (LRRK2) and α-synuclein. In addition, tau protein and glucocerebrosidase (GBA) were also expressed in differentiated neurons (Figure S5). Overall, hiPSC-derived mature dopaminergic neurons expressed a wide range of PD-related proteins to accurately model preferential neuronal vulnerability.

### Differentiated Dopaminergic Neurons are Functional

**Figure 3:**
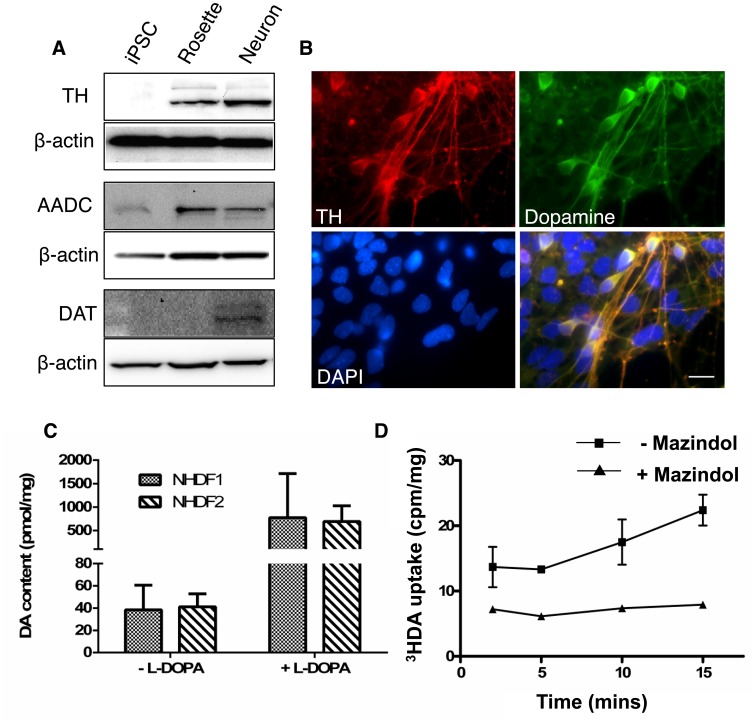
Functional dopamine synthesis and homeostasis in differentiated neuronal cultures. A) Western blot analysis of proteins involved in dopamine synthesis and homeostasis in cells at different stages of maturation (EB: day 0, Rosette: day 16, Neuron: day 35). hiPSCs do not express TH, DAT or AADC but these were expressed as the cells differentiated in culture. DAT is expressed only in differentiated neurons. B) Immunostaining for dopamine in differentiated neurons. This labelling co-localised with TH expression. Scale bar: 70 µm. C) Dopamine content of differentiated neurons was measured by HPLC. Differentiated neurons (35 days total in culture) produced dopamine (48.98 pmol/mg ±0.389) and were responsive to L-DOPA (933.14 pmol/mg ±220.71), indicating high AADC activity. Data shown are from 4–6 wells each of 2 independent experiments and is expressed as mean ± SEM. D) Functional dopamine transporter activity was assessed by ^3^HDA uptake in differentiated neurons. DAT-specific uptake was demonstrated by inhibition by mazindol. Data are expressed as mean ± SEM.

Having demonstrated that differentiated neuronal cultures expressed genes and proteins typical of mature mDA neurons, we next investigated the dopaminergic function of these cells. As differentiation proceeds, the enzymes involved in dopamine synthesis (TH, amino acid decarboxylase [AADC] and DAT) were detected (Figure 3A).

Large clusters of cells co-labelled for dopamine and TH, suggesting that these cells were synthesising dopamine (Figure 3B). To confirm this, high performance liquid chromatography (HPLC) was performed (Figure 3C). Dopamine was detected in both differentiated cell lines. Incubation of neurons with L-3,4-dihydroxyphenylalanine (L-DOPA), the precursor of dopamine, greatly increased dopamine levels, indicating high AADC activity. Furthermore, dopamine was detected in the culture medium (NHDF1: 5.6±0.33 pmol/ml and NHDF2: 8.73±0.90 pmol/ml; data not shown). Functional DAT activity was demonstrated using ^3^H-DA uptake which could be blocked by mazindol, the selective DAT inhibitor (Figure 3D). These mature differentiated cultures of dopaminergic neurons are therefore able to synthesise, release and take up dopamine.

### Physiological Properties of Differentiated Neurons during Maturation

**Figure 4:**
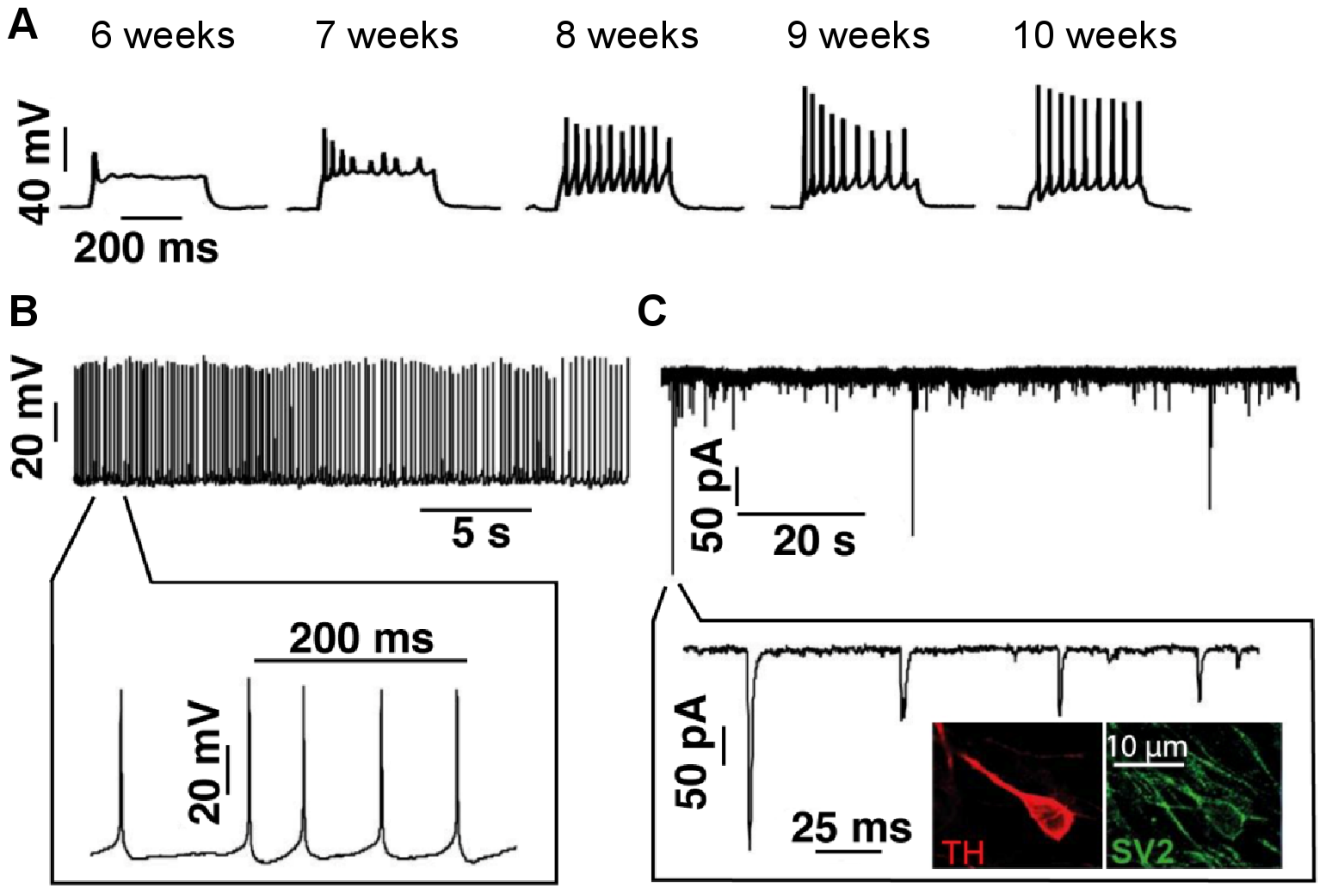
Functional characterisation of the maturation of human midbrain neuronal cultures. (A) Representative current-clamp recordings (400 ms current pulses and inj 40–50 pA) from hiPSC-derived neurons at specified time points (from left panel: 6, 7, 8, 9, and 10 weeks in culture). Neurons show changes in resting membrane potentials reflecting maturation (6 weeks: ∼ −35–40 mV, 10 week: ∼ −65–70 mV). These were recorded in neurons that were not showing pace-making activity. (B) Representative pace-making firing traces in whole-cell patch current clamp configuration and (C) representative traces of the spontaneous EPSCs recorded from 10 week old neuronal cultures (n >6 neurons showed spontaneous pace-making activity). Inset shows immunostaining on fixed cells of co-expression of TH and synaptic vesicle protein, SV2 in differentiated neurons. Similar recordings were obtained from NHDF-1 and -2 lines.

The electrophysiological maturation of hiPSC-derived neuronal cultures over time was examined *in vitro*. One of the clear changes observed as neuronal cultures matured was their ability to fire action potentials repetitively during a 400 ms depolarizing current step (n >6 at each time point measured). None of the neurons at earlier stages of maturation (Figure 4A) could produce more than a single action potential. The spike frequency during depolarizing current steps continued to increase up until 10 weeks of maturation (no further time point measured). Over time, neurons acquired the ability to fire a train of action potentials (Figure 4A). Although within mature neuronal cultures, cell-cell variability was seen (data not shown), the spike frequency upon depolarizing current steps continued to increase. Importantly, we successfully recorded from mature dopaminergic neurons that acquired the maximum frequency of action potential firing as well as showing autonomous slow pace-making activity <10 Hz (Figure 4B; 10 weeks from induction of differentiation at day 0; n >6 neurons). Furthermore, these neuronal cultures displayed spontaneous synaptic activities (Figure 4C).

A selection of whole cell patched neurons were analysed post-hoc in order to determine the presence of TH+ neurons in our samples. We observed ∼22% of biocytin-labelled neurons were TH+ (Figure S4) which is in close alignment with the quantification of total TH+ neurons in our neuronal cultures (Figure 2E).

### Ca^2+^ Signalling during Neuronal Maturation

Ca^2+^ plays a vital role in neuronal physiology, with dyshomeostasis being strongly linked to neurodegenerative diseases [25], [26]. In rodent models of Parkinson’s disease, a critical role for the L-type Ca^2+^ channels in the cellular function of SNc DA neurons has been identified [27], in which Ca^2+^ entry by the activation of L-type Ca^2+^ channels contributes to slow oscillatory potentials that modulate the autonomous pace-making of these midbrain neurons [3].

**Figure 5:**
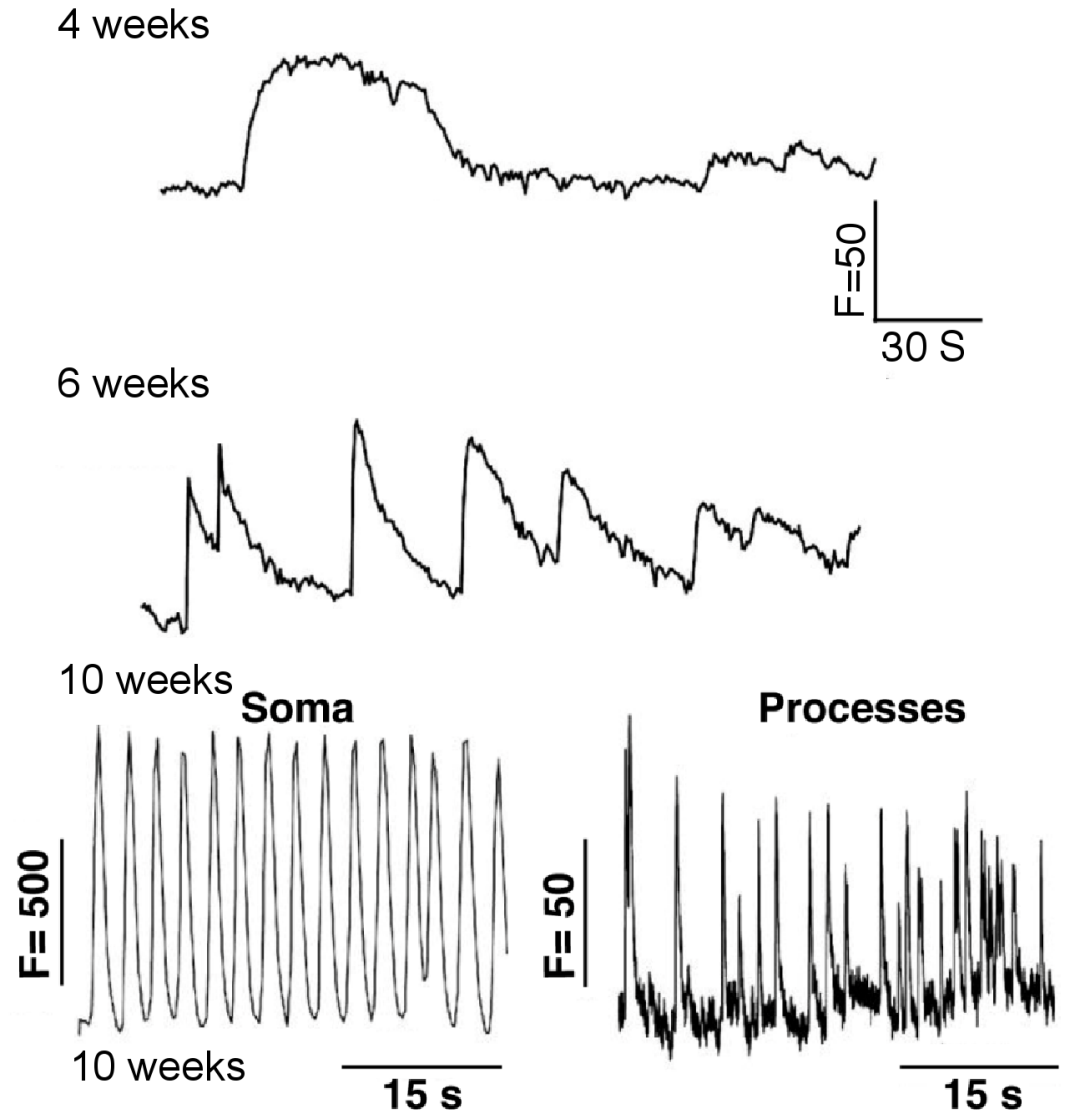
Dynamic changes in Ca^2+^ signals during neuronal maturation. Traces show recordings of spontaneous changes in Ca^2+^ signals after induction of neuronal differentiation from hiPS cells into dopaminergic neurons. Progressive changes in their kinetics were observed, with increase rhythmicity apparent from 6 weeks and onwards in differentiation. By 8 weeks in culture, oscillatory activity typical of dopaminergic neurons was observed in both soma and in the neuronal processes (n >6, for each timepoint). Recordings were obtained from NHDF-1 and -2 lines and gave similar results.

In the light of critical role of Ca^2+^ signalling in Parkinson’s disease, we next recorded changes in spontaneous Ca^2+^ signals during neuronal maturation. Neurons loaded with Fluo-4-AM exhibited slow and weak Ca^2+^ transients in the early stage of maturation (Figure 5, 4 weeks differentiation), after which Ca^2+^ signals became sharper and larger (Figure 5, 6 weeks onwards). At this stage, these spontaneous Ca^2+^ signals (Figure 5, 8 weeks; Video S1) were most likely dependent on Ca^2+^ influx through the plasma membrane. In rodent models, SNc DA neurons display Ca^2+^ oscillations in both the soma and in processes [3]. Spontaneous oscillatory Ca^2+^ signals (Figure 5, 10 weeks; Video S1) were also observed in our human model system in both the soma and the neuronal processes, which were eliminated by acute removal of extracellular Ca^2+^ strongly indicating that these signals are initiated through the plasma membrane, most likely via voltage-dependent Ca^2+^ channels (Figure 5, 8 weeks).

**Figure 6:**
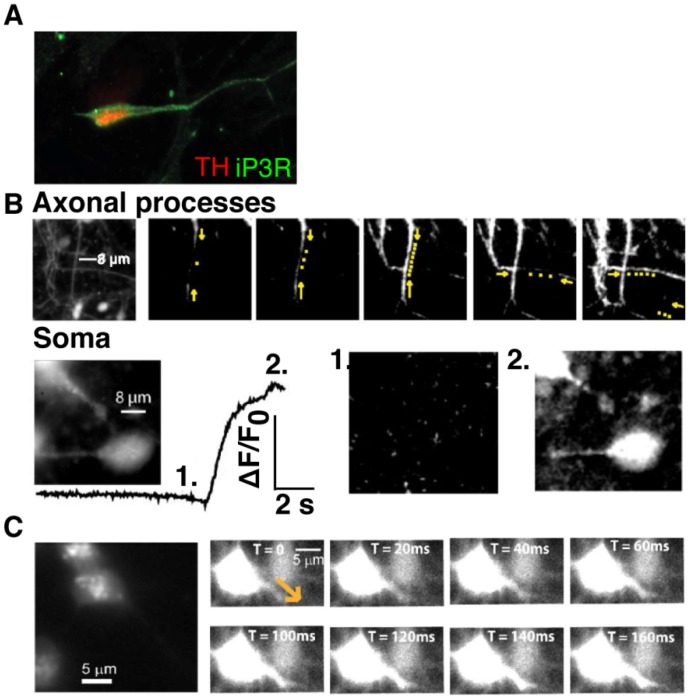
Spontaneous intracellular calcium signals in human hiPS cell-derived neurons. (A) Immunostaining in fixed TH-positive neurons (red) expressed inositol tri-phosphate receptors (IP_3_Rs) in both the soma and in dendritic processes (green). Live cell imaging showed that IP_3_-BM-evoked calcium signals were detected differentiated neurons, both in the processes (B, upper montages) and also from the soma (B lower panels). Yellow puncta in the upper montages represent a time-dependent fluorescence change which indicated propagation of the calcium wave through neuronal processes. The fluorescence profile in the lower panel shows the fluorescence change obtained from the soma (1. before and 2. after elevation of calcium concentration). (C) Left panel indicates the typical distribution of LysoTracker-sensitive acidic organelles in differentiated neurons. Right-hand images illustrate that the propagation of calcium waves upon application of NAADP/AM originated from the neuronal soma toward a dendritic process. Recordings were obtained from 2 different lines (n >3).

### Release of Calcium from Intracellular Calcium Stores

During the earlier stages of neuronal maturation, we observed spontaneous Ca^2+^ signals evoked from intracellular Ca^2+^ stores (Fig. 5, 6 weeks). The endoplasmic reticulum (ER) is the largest intracellular Ca^2+^ store, and expresses inositol 1,4,5-trisphosphate (IP3) receptor Ca^2+^-releasing channels (IP3R). The binding of the second messenger, IP3, to IP3Rs leads to liberation of Ca^2+^ ions from the ER. This evoked Ca^2+^ signalling pathway has been implicated in the modulation of the excitability of midbrain dopaminergic neurons [28]. Thus, we next examined expression of IP3Rs in mature neurons (<8 weeks maturation). Figure 6A shows the distribution pattern of IP3Rs. IP3Rs are expressed throughout the TH+ dopaminergic neuron including the neuronal processes. Consistent with their distribution pattern, the application of the cell-permeant analogue of IP3 (IP3/BM) evoked Ca^2+^ release, not only from neuronal processes, but also from the soma (Figure 6B). Figure 6B upper montages shows Ca^2+^ wave propagation from apical and distal processes meeting, and the lower panels illustrates large Ca^2+^ transients observed in the cell body. We also examined the distribution pattern of lysosomes in our model system. Figure 6C shows lysosomes stained with LysoTracker™-Red. Ample evidence indicates that nicotinic acid adenine dinucleotide phosphate (NAADP) mobilises Ca^2+^ predominantly from lysosomes [29], [30]. Consistent with the distribution pattern of Lysotracker-positive lysosomes, application of NAADP-AM induced Ca^2+^ signal originating from the soma and progressively propagated through neuronal processes (Figure 6C).

### Study of Mitochondria in DA Neurons

**Figure 7:**
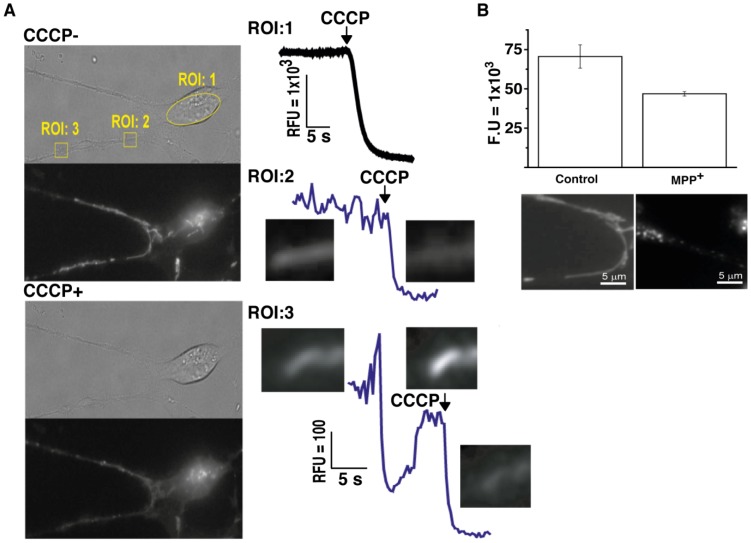
Measurements of mitochondrial membrane potentials and their sensitivity to MPP^+^. (A) Differentiated human neuronal cultures were loaded with mitochondrial membrane indicator TMRM. Changes in fluorescence were measured before (left upper) and after (left lower) application of the membrane potential uncoupler, CCCP. Right panels show quantification of fluorescence changes obtained from the 3 regions of interest marked in the left panels. Induced and spontaneous fluctuations in membrane potential were observed in some cells and changes could be detected at the level of a single mitochondrion (ROI 3). (B) Upper graph represents mean mitochondrial resting potential measured from cell bodies and the effect of exposure to the dopaminergic neuron toxin MPP^+^ for 24 h. Mean resting fluorescence intensity was significantly lower after MPP^+^ treatment (control, F.U. 68.8±13.4×10^3^ and 40 µM MPP+, F.U. 43.6±2.9×10^3^, t <0.05, n >40). Lower panels indicate mitochondrial morphological changes following MPP+ treatment.

Mitochondrial dysfunction has been implicated as an important factor in the pathogenesis of sporadic and familial PD which ultimately leads to apoptotic death of midbrain dopaminergic neurons [31]. Understanding mitochondrial membrane potential dynamics is particularly important, since ATP synthesis is driven by a membrane potential across the inner mitochondrial membrane, which is linked to production of reactive oxygen species (Matsuda Satoru 2013, Oxidative Medicine and Cellular Longevity). Figure 7A shows mitochondria loaded with tetramethylrhodamine- methyl ester (TMRM). Quantification of mitochondrial membrane potential upon application of CCCP indicates immediate depolarization of the mitochondrial membrane potential. Furthermore, we also successfully imaged spontaneous changes in membrane potential at the level of a single mitochondrion in live human neuronal cultures (Figure 7A). The neurotoxin 1-methyl-4-phenylpyridine (MPP^+^) is selectively transported by DAT, which is expressed exclusively in dopaminergic neurons [32]. Neurons derived from hiPS cells showed increased susceptibility to MPP+, as illustrated by reduced mitochondrial membrane potential (Figure 7B). Furthermore, MPP^+^ treatment also resulted in mitochondrial fragmentation (Figure 7B), an indicator of mitochondrial oxidative stress [33].

## Discussion

Here we describe the physiological characterisation of the maturation of hiPSC-derived dopaminergic neuronal cultures. We produced a number of reprogrammed colonies, of which three were selected for rigorous analyses. To analyse genome integrity we compared SNP analysis with M-FISH to assess, albeit on a small-scale, the relative sensitivity of the two methods. Illumina CytoSNP analysis allowed interrogation of 300,000 SNPs throughout the genome, giving a much higher resolution than M-FISH, which can only detect gross changes in karyotype. iPS-NHDF-6 was identified as abnormal by the Illumina CytoSNP analysis, although it should be noted that a completely balanced translocation would not be detected by this method. SNP analysis has been used in previous hiPSC studies to analyse sub-microscopic genomic changes [34]. We suggest therefore, that SNP analysis is a simple, accurate and high throughput method for assessing hiPSC lines.

Pluripotency was assessed in iPS-NHDF-1 and-2 by immunocytochemistry of undifferentiated colonies and of *in vitro* differentiated cells, in combination with more quantitative transcriptome analysis using the PluriTest [18]. Traditionally, the gold standard for assessing pluripotency in hiPSCs has been the generation of tissues reminiscent of the three germ layers within a teratoma, as judged by visual histological assessment. We consider this unnecessary in light of novel transcriptional analyses. Teratoma formation studies are time-consuming, costly, require large numbers of animals and are not suitable for research programs generating large numbers of hiPSC lines. In addition, teratoma assays are primarily qualitative and difficult to standardize [35]. The PluriTest uses a large comprehensive database of genome-wide transcriptional profiles from many different hESC and hiPSC lines, plus data from differentiated cell types and from developing and adult tissue. This system also benefits from being able to distinguish from fully and partially reprogrammed cells, which the teratoma assay is not able to do [36]. Microarray-based analysis of gene expression is relatively low-cost and accessible to most laboratories, therefore the PluriTest offers an economical, quantitative alternative to teratoma formation.

The use of Y27632 for preventing cell detachment-related cell death and increasing cell survival has been well documented in stem- and progenitor-cell populations [37], [38]. We applied these principles to our differentiation procedure and found that supplementing our differentiation medium with Y27632 increased the expansion of our neural progenitor colonies, therefore increasing the yield of differentiated neurons.

Directed differentiation of both NHDF-1 and -2 into dopaminergic neurons was successful and with similar high efficiencies in both lines consistent with data from previous publications [20], [39]. In order to determine the maturity of our differentiated cultures, we examined alternative splicing of *MAPT* exon 10. *MAPT* splicing has previously been studied in hESC-derived neurons which express all 6 isoforms, with more exon 10^+^ tau protein being expressed as cells mature *in vitro* [40]. We found in our model system that exon 10^+^ *MAPT* was not expressed in undifferentiated hiPSCs but was expressed in mature differentiated neurons with expression similar to that in adult neurons from post-mortem brain. By examining *MAPT* exon 10^+^ splicing it is possible to distinguish between foetal and adult neuron expression patterns. Previous reports have suggested that the gene expression profile of hESC-derived differentiated neurons resembled a more foetal rather than adult state [41]. It is not clear, however, how long the neurons were matured in culture in that study. From the *MAPT* splicing data, and the maturation of synaptic activity over time that we observe, we conclude that our hiPSC-derived neuronal cultures contain a representative population of functionally mature adult cells.

Analysis of gene and protein expression revealed that these neuronal cultures expressed markers of midbrain dopaminergic neurons, including the SNc A9 marker, GIRK2, suggesting that this population contains the vulnerable cell type in PD [42], [43]. Quantification of these neurons revealed that over half (53.5%) of the TH+ DA neurons expressed GIRK2, therefore were of the A9 phenotype. Sub-populations of neurons were identified in which clusters of FOXA2 positive cells were identified (37% of total cells). Upon quantification of differentiation we found 29.8% of TH+ cells were also FOXA2+, which are analogous to floorplate-derived ventral DA neurons, which is comparable to previous reports [21], [44]. The expression of proteins related to PD, including Tau, LRRK2, GBA and α-synuclein, demonstrates the potential of patient-derived hiPSCs to be utilised for the analysis of mechanisms underlying preferential neuronal vulnerability in PD. Indeed several studies have begun to uncover cellular phenotypes in PD-derived hiPSCs [9, 11, 12, [45], 42]. The expression of proteins related to dopamine homeostasis was supported by demonstrating their ability to synthesise, release and uptake dopamine. Many laboratories have performed characterisations of differentiated human dopaminergic neurons from embryonic and induced stem cells [39], [46]–[48] but we wished to study dopamine homeostasis further and found that differentiated neurons also demonstrated functional DAT, by use of ^3^H-DA, a technique used only a very few times to assess DAT function in dopaminergic neurons differentiated from mouse ESCs and hiPSCs *in vitro* [49], [50]. Together, these assays will be useful for analysis dopamine homeostasis in human models of PD.

We sought to comprehensively characterise the electrophysiological properties of neurons as they mature in culture. Several other protocols have been reported which generate functional dopaminergic neurons from ESCs and hiPSCs [10], [39], [46], [48], [51], [52] and in this report we successfully recorded functional properties of human DA neurons as they mature in culture. Data from human dopaminergic neurons is limited although some developmental data has been obtained by recording from human foetal mesencephalic grafts that have been transplanted into rats. Extracellular recordings from these cells showed typical bi- or triphasic action potentials with a long duration (2–5 ms) and spontaneous activity [53]. We observed a similar pattern in population of our hiPSC-derived neurons, and as neuronal cultures were matured over time, they also exhibited a typical pace-making firing pattern characteristic of SNc DA neurons in rodent models (Figure 4B) [54]. Whole cell patch clamp experiments in our cultures showed that as these cells matured *in vitro*, their ability to fire trains of multiple action potentials increased. This, together with the existence of EPSPs, is in accordance with data obtained from postnatal rat nigrostriatal neurons whereby spontaneous burst activity was initially found at low frequency but as maturation proceeded, the mean firing rate increased until pacemaker-like activity was observed. Maximal adult values were achieved within 4 weeks post-natal in rat neurons, therefore these data support the *in vitro* maturation of dopaminergic pace-making activity in our cultured neurons after 10 weeks of maturation in culture [55]. Note that adult gene expression patterns were observed in our neuronal cultures after 4 weeks of differentiation (MAPT exon slicing, figure 2G). However, mature physiological behaviour was not exhibited until much later in maturation (6–8 weeks in culture; Figures 4 and 5).

Data from rodent mDA neurons and from computational models have indicated a critical role for L-type Ca^2+^ channels in driving the pacemaking activity of these cells [56]–[58]. It has recently been shown these channels support, but are not necessary for, autonomous pacemaking activity [3], although this remains controversial. A study of mouse ES-derived dopaminergic neurons has shown increased frequency and amplitude of spontaneous calcium oscillations in neurons as they mature [59]. We observed a similar pattern of maturation in our human neurons. Although there is a difference in the developmental time scale (mouse vs human), we observed a similar pattern of development in spontaneous Ca^2+^ signals. Furthermore, autonomous pace-making calcium influx was observed in both the neuronal soma and processes in mature human neurons, which is highly characteristic of SNc dopaminergic neurons [3]. Here we have demonstrated that hiPSC-derived DA neurons are excitable and that they can produce calcium-dependent action potentials. These neurons undergo functional development over time, during which they exhibit differential signatures of spontaneous Ca^2+^ signals, utilising extra- and intra- cellular Ca^2+^ stores. Many reports have demonstrated the importance of Ca^2+^ regulation in neurodegenerative diseases. SNc DA neurons exhibit autonomous pace-making activity which is supported by L-type Ca^2+^ channels [60], [61]. Ca^2+^-dependent DA synthesis is greater in SNc DA neurons comparing to those in ventral tegmental area [62]. In addition, SNc DA neurons express lower levels of the Ca^2+^ buffering protein, calbindin. These characteristic features observed in rodent models are important when modelling PD pathophysiology in human DA neurons derived from hiPS cells.

Finally, we demonstrate that this human dopaminergic neuronal model can be used to study mitochondrial homeostasis in the presence of MPP+. The Complex I inhibitor, MPP+ is widely used as a specific toxin for midbrain DA neurons. Injection of the precursor, MPTP, into the primate brain recapitulates most of the clinical and pathological features of PD, including selective and specific degeneration of the midbrain DA neurons [63]. MPP^+^ enters cells via the dopamine transporter, DAT. Previous studies have shown that MPP+-insensitive cell lines, such as COS cells, can become sensitised by the forced expression of DAT [64]. Therefore, we assume that the cells affected my MPP+ in these experiments are expressing DAT, and therefore DA neurons. hiPSCs have previously been generated from PD patients with Parkin [10] and PINK1 [13] mutations and the potential to model mitochondrial metabolism and dynamics has also been demonstrated [65]. By studying mitochondria in fully functioning human dopaminergic neurons we may be able to elucidate their involvement in the pathological process of PD.

## Summary

Here we offer the first comprehensive analysis of the physiology of cultures of human dopaminergic neurons differentiated from hiPSCs. We have shown that these cells have the physiological hallmarks of mDA neurons previously only defined from rodent slice preparations and that this makes them an excellent model system by which to study human neuronal physiology. The potential to apply these reprogramming and differentiation protocols to patient derived cells can now be realised. We believe that by fully characterising the physiological function of these cells we have a reliable model for neuronal development and disease modelling, particularly for PD.

## Supporting Information

Figure S1 **Further characterisation of hiPSC lines.** A) RT-PCR for expression of endogenous pluripotency genes once reprogramming had been completed: Oct, Sox, Klf, Myc and Nanog. cDNA template for each hiPSC line (‘C’). Negative controls consisted of RNA template (‘R’) for each hiPSC line; no template (H_2_O); Plasmid = pMXS+exogenous version of the relevant pluripotency gene. The hESC line, HUES2 serves as a positive control; the parental fibroblast line, NHDF, is also shown. B) qRT-PCR to assess silencing of the 5 exogenous transgenes in the derived hiPSC lines, relative to NHDF+Y (NHDF infected with all 5 reprogramming vectors then harvested at day 5); parental, uninfected NHDF are also shown. C) DNA Fingerprinting using PowerPlex® 16 HS System for detection of sixteen loci to confirm that the lines iPS-NHDF-1 and iPS-NHDF-2 derive from the parent fibroblasts NHDF.
(TIF)

Figure S2 **Rock inhibitor increases NPC proliferation.** Embryoid bodies were plated on Geltrex-coated dishes in medium either in the presence or absence of Rock inhibitor (Y27632). Images were taken 4 days later and show dramatically increased area of cell growth in the presence of Rock inhibitor compared with EBs grown in its absence. Scale bar: 250 µm.
(TIF)

Figure S3 **Further quantification of midbrain DA neuronal cultures.** A) Cell counts were performed using immunostaining for GIRK2 and TH in order to quantify A9 dopaminergic neuronal differentiation. A large proportion of the cells expressed GIRK2, but the total number of GIRK2+TH-positive cells was relatively low in comparison to the entire cell population. However, over half of the cells which expressed TH also expressed GIRK2, indicating a large proportion of our differentiated midbrain DA neurons are of the A9 phenotype. B) Similar analysis was carried out for FOXA2 and it was again found that a large proportion of the TH+ neurons were also FOXA2 positive.
(TIF)

Figure S4 **Post-hoc identification of TH+ neurons following whole-cell patch clamp experiments.** In order to demonstrate the presence of TH+ neurons in whole cell patch clamp experiments, a sample of neurons was post-hoc labelled. The recording electrode was filled with biocytin which diffused into the patched cell during recording. Neuronal cultures were fixed and stained for TH (green) and biocytin is shown in red. * indicates a successfully patched TH+ neuron. From this sample, 22.2% of patched cells were TH+.
(TIF)

Figure S5 **Expression of Parkinson’s disease-related proteins in differentiated midbrain neurons.** Immunostaining of differentiated neurons shows that proteins that have been implicated in the pathology of Parkinson’s disease are expressed in these cells. A: α-synuclein; B: LRRK2; C: Tau; D: GBA. Scale bars: 20 µm.
(TIF)

Table S1 RT-PCR primers used for characterising differentiated hiPSC cultures.
(DOC)

Table S2 Karyostudio Detected Regions Report for NHDF Lonza and derived hiPSc lines. Detected Regions autosomal differences between NHDF Lonza parental fibroblasts and derived hiPSC lines are reported here.
(DOC)

Methods S1 **Further Characterisation of iPSC lines.** Methods for assessing the activation/silencing of pluripotency genes and for DNA fingerprinting.
(DOC)

Video S1 **Mature spontaneous Ca^2+^ signals recorded from differentiated neurons.** Ca^2+^ signals were observed in mature neuronal cultures (>8 weeks differentiation) and are most likely associated with action potential firing. Autonomous pacemaking Ca2+ firing was observed in both the neuronal processes and the cell bodies in these neurons.
(WMV)
