# Supplementary figures and images for: Physiological Characterisation of Human iPS-Derived Dopaminergic Neurons
Source: PLoS One. 2014 Feb 21;9(2):e87388. doi: 10.1371/journal.pone.0087388 (PMC3931621; doi:10.1371/journal.pone.0087388)

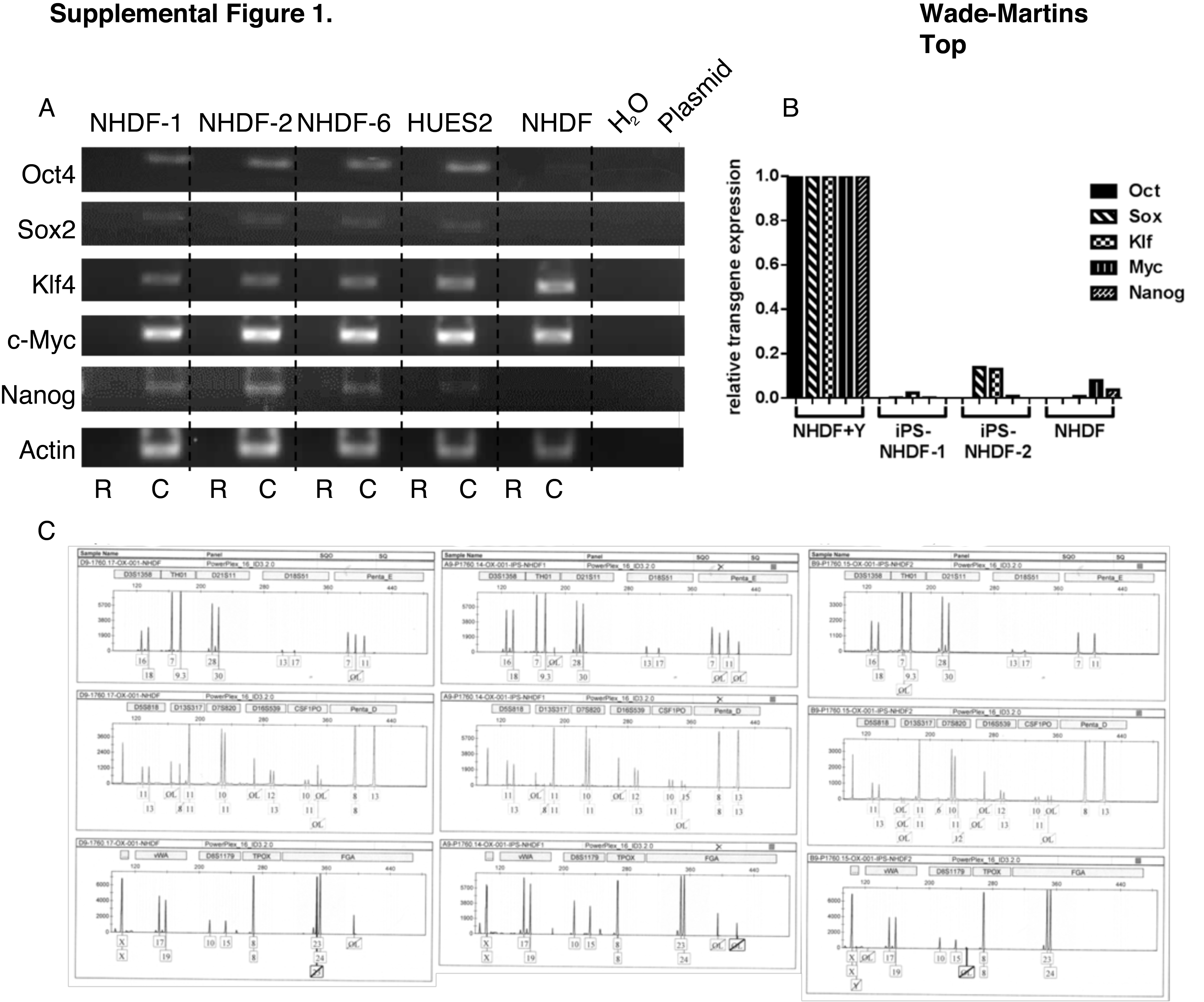

Supplement: Figure S1 — Further characterisation of hiPSC lines. A) RT-PCR for expression of endogenous pluripotency genes once reprogramming had been completed: Oct, Sox, Klf, Myc and Nanog. cDNA template for each hiPSC line (‘C’). Negative controls consisted of RNA template (‘R’) for each hiPSC line; no template (H2O); Plasmid = pMXS+exogenous version of the relevant pluripotency gene. The hESC line, HUES2 serves as a positive control; the parental fibroblast line, NHDF, is also shown. B) qRT-PCR to assess silencing of the 5 exogenous transgenes in the derived hiPSC lines, relative to NHDF+Y (NHDF infected with all 5 reprogramming vectors then harvested at day 5); parental, uninfected NHDF are also shown. C) DNA Fingerprinting using PowerPlex® 16 HS System for detection of sixteen loci to confirm that the lines iPS-NHDF-1 and iPS-NHDF-2 derive from the parent fibroblasts NHDF. (TIF) [file pone.0087388.s001.tif]

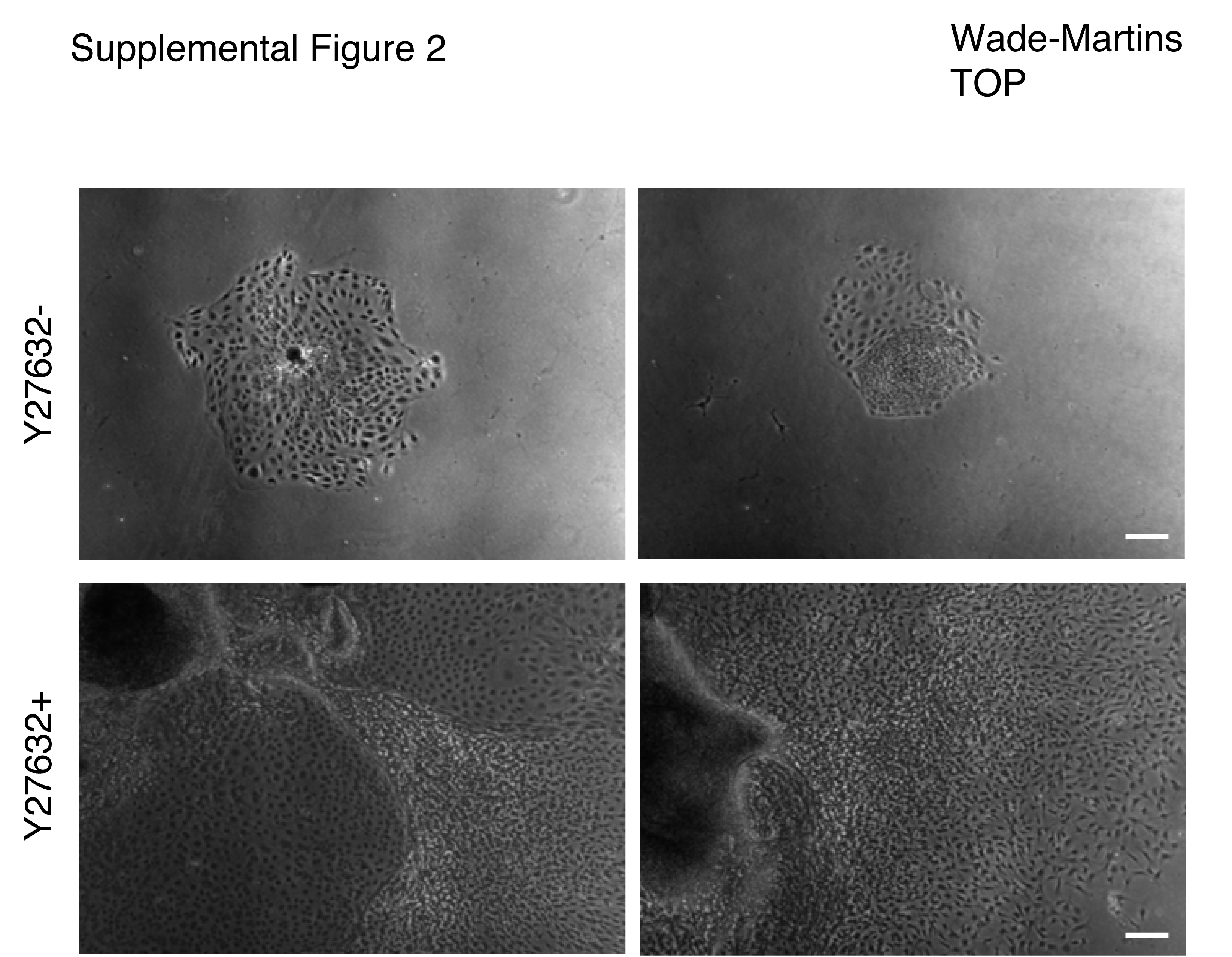

Supplement: Figure S2 — Rock inhibitor increases NPC proliferation. Embryoid bodies were plated on Geltrex-coated dishes in medium either in the presence or absence of Rock inhibitor (Y27632). Images were taken 4 days later and show dramatically increased area of cell growth in the presence of Rock inhibitor compared with EBs grown in its absence. Scale bar: 250 µm. (TIF) [file pone.0087388.s002.tif]

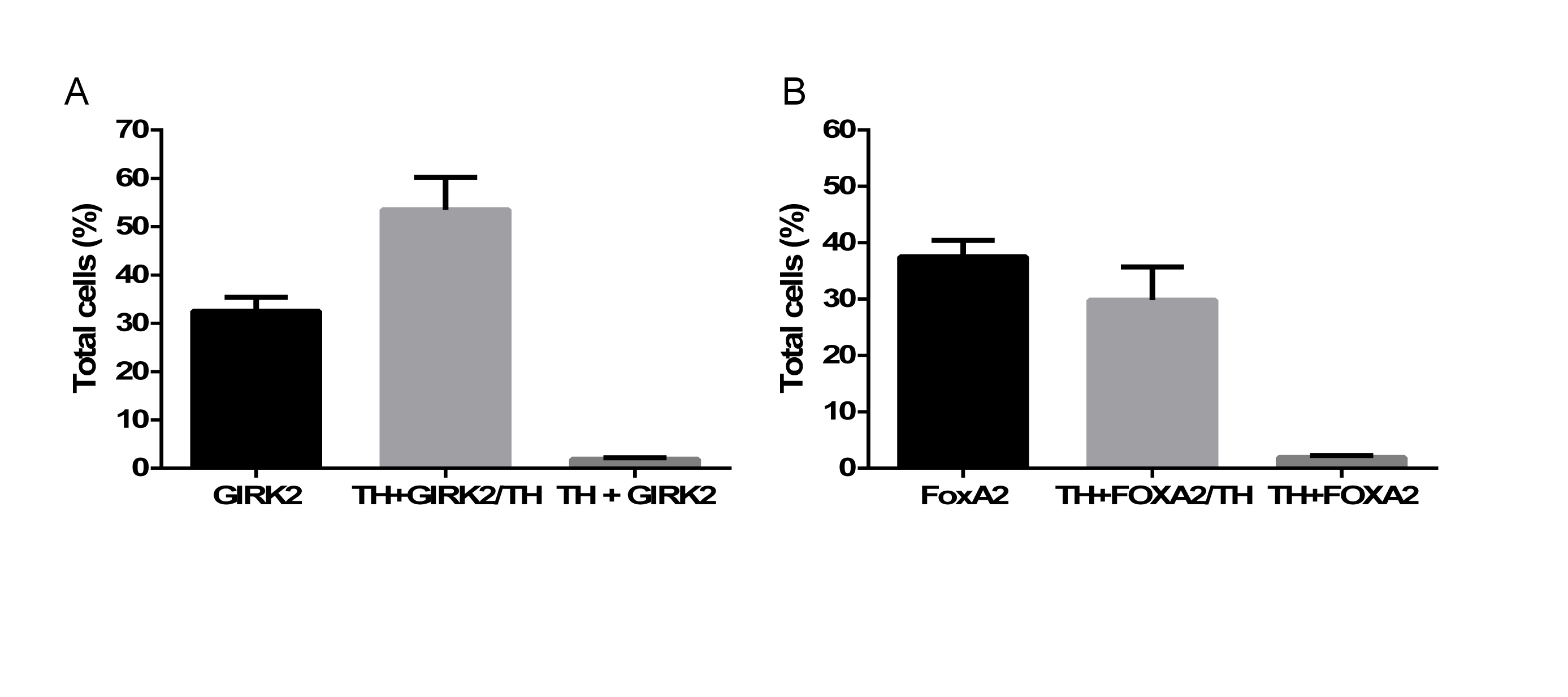

Supplement: Figure S3 — Further quantification of midbrain DA neuronal cultures. A) Cell counts were performed using immunostaining for GIRK2 and TH in order to quantify A9 dopaminergic neuronal differentiation. A large proportion of the cells expressed GIRK2, but the total number of GIRK2+TH-positive cells was relatively low in comparison to the entire cell population. However, over half of the cells which expressed TH also expressed GIRK2, indicating a large proportion of our differentiated midbrain DA neurons are of the A9 phenotype. B) Similar analysis was carried out for FOXA2 and it was again found that a large proportion of the TH+ neurons were also FOXA2 positive. (TIF) [file pone.0087388.s003.tif]

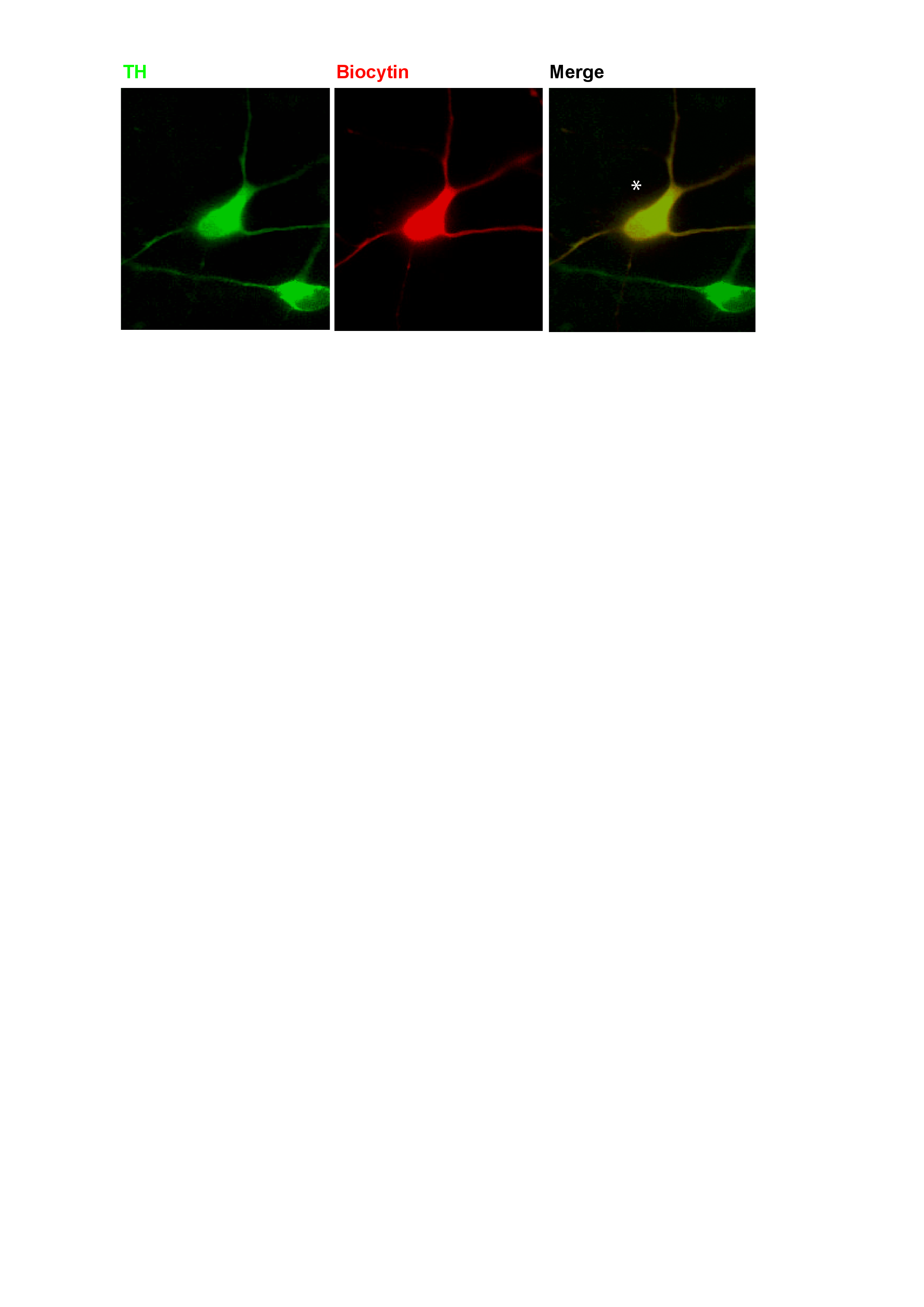

Supplement: Figure S4 — Post-hoc identification of TH+ neurons following whole-cell patch clamp experiments. In order to demonstrate the presence of TH+ neurons in whole cell patch clamp experiments, a sample of neurons was post-hoc labelled. The recording electrode was filled with biocytin which diffused into the patched cell during recording. Neuronal cultures were fixed and stained for TH (green) and biocytin is shown in red. * indicates a successfully patched TH+ neuron. From this sample, 22.2% of patched cells were TH+. (TIF) [file pone.0087388.s004.tif]

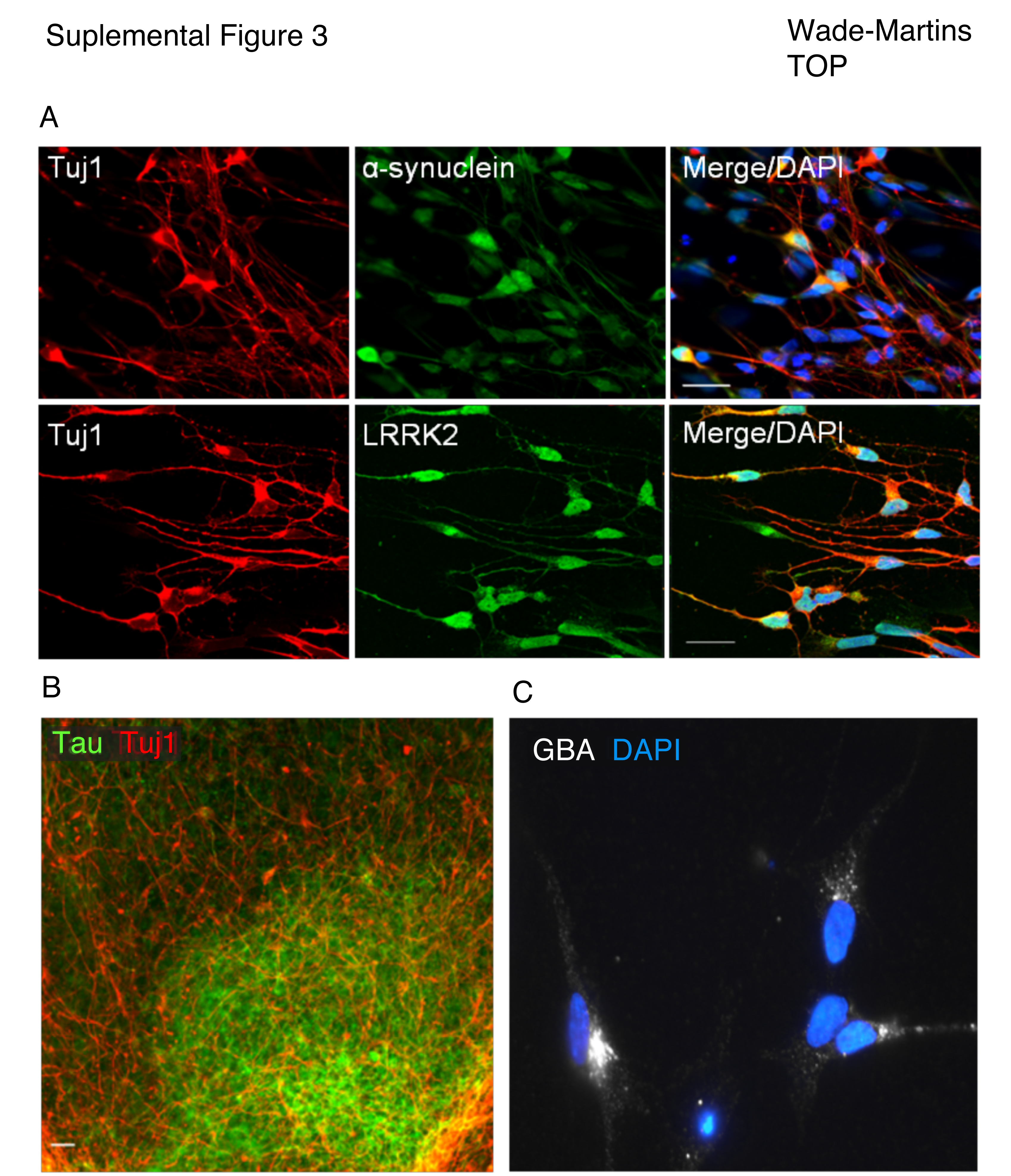

Supplement: Figure S5 — Expression of Parkinson’s disease-related proteins in differentiated midbrain neurons. Immunostaining of differentiated neurons shows that proteins that have been implicated in the pathology of Parkinson’s disease are expressed in these cells. A: α-synuclein; B: LRRK2; C: Tau; D: GBA. Scale bars: 20 µm. (TIF) [file pone.0087388.s005.tif]
