# Supplementary material for: Physiological Characterisation of Human iPS-Derived Dopaminergic Neurons
Source: PLoS One. 2014 Feb 21;9(2):e87388. doi: 10.1371/journal.pone.0087388 (PMC3931621; doi:10.1371/journal.pone.0087388)
Supplement: Table S1 — RT-PCR primers used for characterising differentiated hiPSC cultures. (DOC) [file pone.0087388.s006.doc]

Supplemental table 1. RT-PCR primers

| Marker | Gene | Forward (5’ – 3’) | Reverse (5’ – 3’) | Product size |
| --- | --- | --- | --- | --- |
| Pluripotency | *SOX2* | AGCTACAGCATGATGCAGGA | GGTCATGGAGTTGTACTGCA | 126 |
| Pluripotency | *OCT4* | CGTTCTCTTTGGAAAGGTGTTC | ACACTCGGACCACGTCTTTC | 320 |
| Neural progenitor | *PAX6* | AACAGACACAGCCCTCACAAACA | CGGGAACTTGAACTGGAACTGAC | 319 |
| Neural progenitor | *NESTIN* | ACCAAGAGACATTCAGACTCC | CCTCATCCTCATTTTCCACTCC | 303 |
| DA transcription factor | *PITX3* | GGACTAGGCCCTACACACAGA | TCCGCGCACGTTTATTTC | 160 |
| DA transcription factor | *NURR1* | CGACATTTCTGCCTTCTCC | GGTAAAGTGTCCAGGAAAAG | 297 |
| DA transcription factor | *EN1* | CTGGGTGTACTGCACACGTTAT | TACTCGCTCTCGTCTTTGTCCT | 357 |
| DA neuron | *GIRK2* | GGAACTGGAAATTGTGGTCAT | CATCACCATTTCTTTCTGTTT | 341 |
| DA neuron | *DAT* | CCCACTACGGAGCCTACATCTT | CAATGGCGTAGGCCAGTTTC | 785 |
| DA neuron | *TH* | GCGGTTCATTGGGCGCAGG | CAAACACCTTCACAGCTCG | 215 |
| Neuron | *TUJ1* | GCCTCTTCTCACAAGTACGTGCCTCG | GGGGCGAAGCCGGGCATGAACAAGTGCAG | 183 |
| Housekeeper | *GAPDH* | GTGGACCTGACCTGCCGTCT | GGAGGAGTGGGTGTCGCTGT | 153 |
| MAPT | Exon 9-11 | GAACCTCCAAAATCAGGGGATCGC | CACCTTGCTCAGGTCAACTGGT |  |
