# Supplementary material for: Physiological Characterisation of Human iPS-Derived Dopaminergic Neurons
Source: PLoS One. 2014 Feb 21;9(2):e87388. doi: 10.1371/journal.pone.0087388 (PMC3931621; doi:10.1371/journal.pone.0087388)
Supplement: Table S2 — Karyostudio Detected Regions Report for NHDF Lonza and derived hiPSc lines. Detected Regions autosomal differences between NHDF Lonza parental fibroblasts and derived hiPSC lines are reported here. (DOC) [file pone.0087388.s007.doc]

| **Supplementary Table 2.** | | |  |  |  |  |  |  |  |  |
| --- | --- | --- | --- | --- | --- | --- | --- | --- | --- | --- |
| **Karyostudio Detected Regions Report for NHDF Lonza and derived iPSc lines.** | | | | | | | | | | |
|  | | | | | | | | | | |
| Sample ID | Chr | Start | Stop | Length | Confidence | Comment | CNV Index | Cytobands | # Markers | Genes |
| NHDF Lonza (parental fibroblasts) | 14 | 20213937 | 20425051 | 211114 | 53 | small amplification, low confidence, 7 genes | 7 | q11.2 | 13 | ANG; RNASE4; ANG; FAM12A; FAM12B; RNASE6; RNASE1; |
| iPS-NHDF-1 | 7 | 100988184 | 101132338 | 144154 | 68 | small amplification, low confidence, 2 genes | 6 | q22.1 | 25 | EMID2; MYLC2PL; |
| iPS-NHDF-2 | 7 | 100971604 | 101132338 | 160734 | 66 | small amplification, low confidence, 2 genes | 7 | q22.1 | 27 | EMID2; MYLC2PL; |
