# Supplementary material for: Physiological Characterisation of Human iPS-Derived Dopaminergic Neurons
Source: PLoS One. 2014 Feb 21;9(2):e87388. doi: 10.1371/journal.pone.0087388 (PMC3931621; doi:10.1371/journal.pone.0087388)
Supplement: Methods S1 — Further Characterisation of iPSC lines. Methods for assessing the activation/silencing of pluripotency genes and for DNA fingerprinting. (DOC) [file pone.0087388.s008.doc]

**Supporting methods**

**Further characterisation of iPSC lines**

RT-PCR for assessing activation of endogenous pluripotency gene expression and QRT-PCR for assessing the degree of silencing of transgene sequences, were carried out using primer sequences published by Takahashi and Yamanaka, supplementary table S12 , except that in place of the published reverse primer (pMXs-AS3200 TTA TCG TCG ACC ACT GTG CTG CTG), we used our own primer, pMXs-AS3200v2 (TTA TCG TCG ACC ACT GTG CTG GCG) which had exactly the same sequence as the pMXs vector and which therefore amplified more efficiently. Also, for amplifying transgene mNanog, the forward primer GCT CCA TAA CTT CGG GGA GG was used.

RNA was extracted from fibroblasts using the RNeasy kit (Qiagen), then reverse transcription was carried out using the RetroScript kit (Ambion), using 2ug template RNA. QRT-PCR was carried out on the StepOne Plus Real Time PCR machine (Applied Biosystems), using SYBR green PCR mix + ROX (Applied Biosystems), and iPS targets were compared to actin B control (actin B primers, Eurogentec) and subsequently to fibroblasts harvested 5 days after infection with the reprogramming vectors .

DNA fingerprinting was carried out to confirm that the iPSC lines were derived from the parental fibroblasts. Genomic DNA was purified using DNA Blood and Tissue kit (Qiagen) and analysed by Complement Genomics ([http://www.compgeno.com](http://www.compgeno.com/)) using the PowerPlex 16 system (Promega) for multiplex identification of 16 genomic loci, and Genemapper IDv3.2.0 software (Applied Biosystems).

1. Takahashi, K., Tanabe, K., Ohnuki, M., Narita, M., Ichisaka, T., Tomoda, K., and Yamanaka, S. (2007). Induction of Pluripotent Stem Cells from Adult Human Fibroblasts by Defined Factors. Cell *131*, 861-872.

2. Livak, K., and Schmittgen, T. (2001). Analysis of Relative Gene Expression Data Using Real-Time Quantitative PCR and the 2−ΔΔCT Method. Methods *25*, 402-408.
